# Crystal structure of (*Z*)-*N*-benzyl­idene-1-phenyl­methanamine oxide hydrogen peroxide monosolvate

**DOI:** 10.1107/S2056989017014499

**Published:** 2017-10-20

**Authors:** Andrei V. Churakov, Petr V. Prikhodchenko, Alexander G. Medvedev, Alexey A. Mikhaylov

**Affiliations:** a Institute of General and Inorganic Chemistry, Russian Academy of Sciences, Leninskii prospekt 31, Moscow 119991, Russian Federation

**Keywords:** crystal structure, peroxosolvate, *N*-oxide, nitrone, hydrogen-bond motif

## Abstract

The title adduct, C_14_H_13_NO·H_2_O_2_, consists of (*Z*)-*N*-benzyl­idene-1-phenyl­methanamine oxide and hydrogen peroxide mol­ecules in a 1:1 ratio. The organic coformer adopts a skew geometry with an inter-aryl-ring dihedral angle of 81.9 (2)°. In the crystal, the organic and peroxide mol­ecules are linked through both peroxide O—H donor groups to oxide O-atom acceptors, giving one-dimensional chains extending along the *b* axis. Present also are weak inter­molecular C—H⋯O hydrogen-bonding inter­actions.

## Abstract 2

The title structure consists of a (*Z*)-*N*-benzyl­idene-1-phenyl­methanamine oxide and a hydrogen peroxide mol­ecule linked through both O—H groups into a one-dimensional chain structure.

## Chemical context   

Peroxosolvates are solid adducts that contain hydrogen peroxide mol­ecules of crystallization in the same manner as the water in crystalline hydrates. Today, some of these are widely used as environmentally friendly bleaching compounds (Jakob *et al.*, 2012 ▸) and oxidizing agents in organic synthesis (Ahn *et al.*, 2015 ▸). Hydrogen bonding in peroxosolvates is of particular inter­est since it may be used for modelling of hydrogen peroxide behaviour in various significant biochemical processes (Kapustin *et al.*, 2014 ▸).
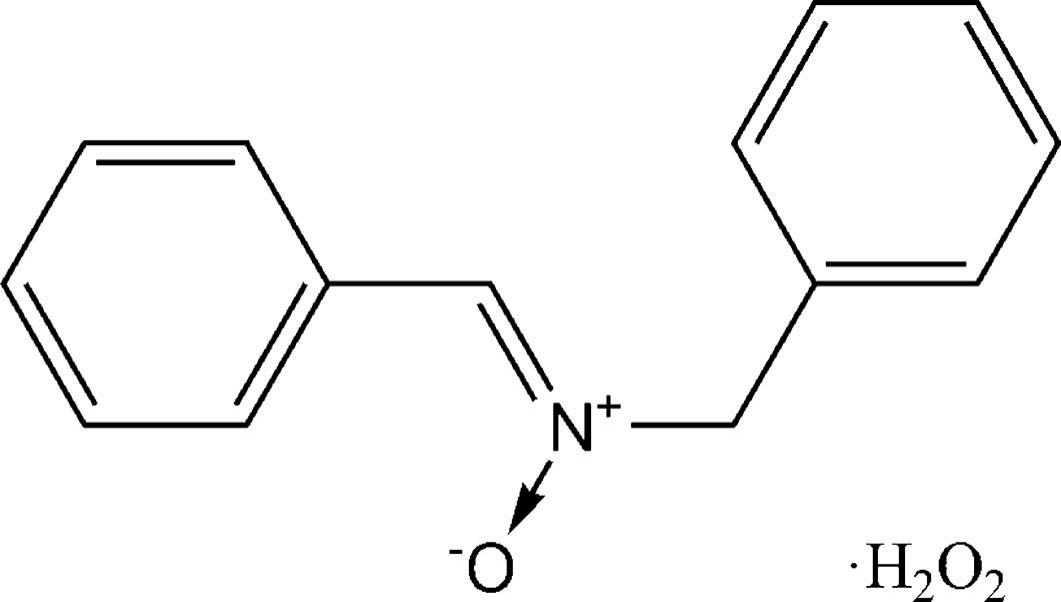


It is known that nitro­nes *R* ^1^–CH=N(O)–*R* ^2^ [*R* ^1^, *R* ^2^ = aryl (Ar) or alkyl (Alk)] are readily available by oxidation of secondary amines using hydrogen peroxide (Goti *et al.*, 2005 ▸). We supposed that the combination of oxidizing and cocrystallizing properties of hydrogen peroxide might afford an opportunity to obtain nitrone peroxosolvates in one step. We prepared the title 1:1 adduct of (*Z*)-*N*-benzyl­idene-1-phenyl­methanamine oxide with hydrogen peroxide and the structure is reported herein.

## Structural commentary   

**Figure 1:**
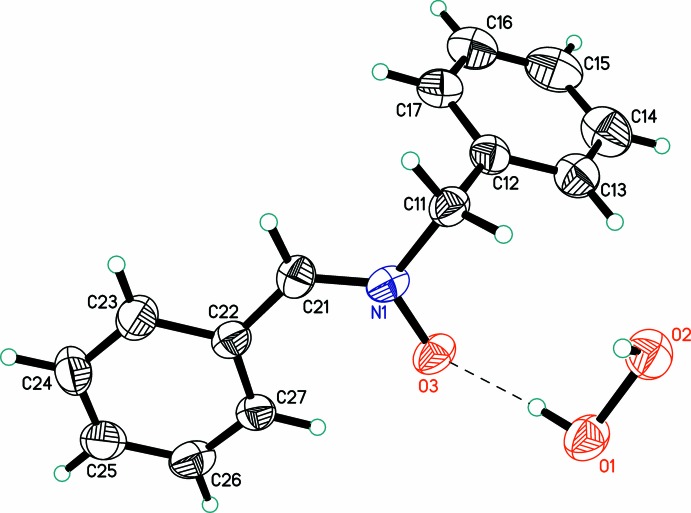
The asymmetric unit in the title structure. Displacement ellipsoids are shown at the 50% probability level and the hydrogen bond is drawn as a dashed line.

In the structure of the title adduct (Fig. 1 ▸), all bond lengths and angles in the organic coformer exhibit normal values for nitrone derivatives (Cambridge Structural Database, Version 5.38, May 2017; Groom *et al.*, 2016 ▸). The nitrone fragment Ph—CH=N(O)—C is planar to within 0.128 (3) Å. It is almost perpendicular to the benzyl substituent C11–C17, with an O3—N1—C11—C12 torsion angle of 72.7 (4)°, and forms a dihedral angle between the two aryl rings of 81.9 (2)°. This is the same conformation as was previously observed in the structure of the pure coformer (Herrera *et al.*, 2001 ▸). The organic mol­ecule forms two hydrogen bonds, involving the negatively charged oxide atom O3, with adjacent peroxide mol­ecules and the conformation is stabilized by an aromatic C27—H⋯O3 hydrogen bond (Table 1 ▸). As expected, the N1—O3⋯O(peroxo) angles are close to trigonal [117.9 (2) and 126.2 (2)°].

In the peroxide mol­ecule, the O—O distance is 1.467 (4) Å. This value is close to those previously observed in the accurately determined structures of crystalline hydrogen peroxide [1.461 (3) Å; Savariault *et al.*, 1980 ▸] and urea perhydrate [1.4573 (8) Å; Fritchie & McMullan, 1981 ▸]. Partial substitutional disorder of hydrogen peroxide by water mol­ecules (Pedersen, 1972 ▸) was not observed in the present structure since no residual peaks with an intensity of 0.14 e Å^−3^ were seen in the hydrogen peroxide mol­ecule region (Churakov *et al.*, 2005 ▸). The H_2_O_2_ mol­ecule lies on a general position and presents a skew geometry, with the H—O—O—H torsion angle equal to 88 (4)°, and forms just two donor hydrogen bonds. It should be noted that the maximum possible number of hydrogen bonds formed by H_2_O_2_ is six (two donor and four acceptor; Chernyshov *et al.*, 2017 ▸).

## Supra­molecular features   

**Figure 2:**
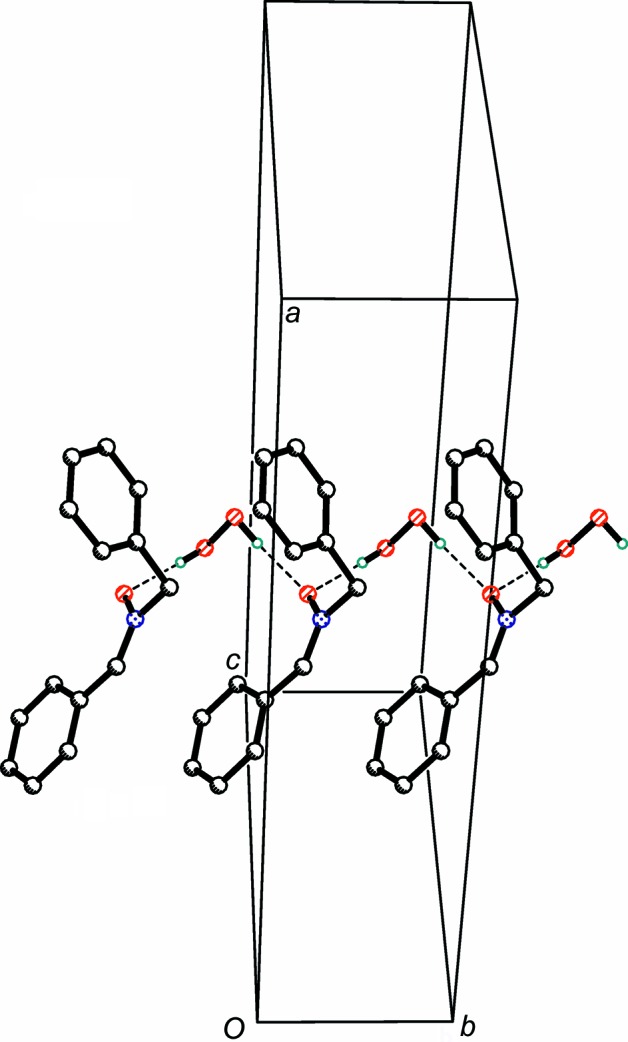
Hydrogen-bonded chains extending along the *b* axis. H atoms on C atoms have been omitted for clarity. Hydrogen bonds are drawn as dashed lines.

In the title crystal, the organic and peroxide mol­ecules are linked into hydrogen-bonded chains extending along the *b* axis through charge-supported moderate HOOH⋯^−^O—N hydrogen bonds, with O⋯O separations of 2.707 (5) and 2.681 (5) Å (Table 1 ▸ and Fig. 2 ▸). Similar chains formed by *N*-oxide and H_2_O_2_ mol­ecules were previously observed in the structure of risperidone *N*-oxide hydrogen peroxide methanol solvate (Ravikumar *et al.*, 2005 ▸). In the present one-dimensional structure, minor weak non-aromatic C—H⋯O(peroxide) hydrogen-bonding inter­actions are also present.

## Database survey   

**Figure 3:**
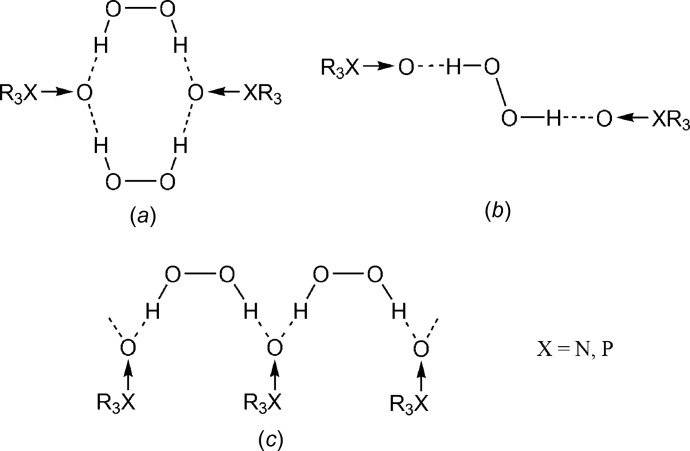
Hydrogen-bonded motifs in the structures of *N*- and *P*-oxides.

The Cambridge Structural Database (Groom *et al.*, 2016 ▸) contains data for nine peroxosolvates of *N*- and *P*-oxides with one or two *R*_3_ *X* ^+^ → O^−^ functional groups (*X* = N, P; *R* = Alk, Ar). It is of inter­est that all of these were obtained by oxidation of the corresponding amines (phosphines) using hydrogen peroxide, followed by immediate crystallization from the reaction mixtures. Analysis of the crystal packing for these compounds reveals three main supra­molecular motifs (Fig. 3 ▸ *a*–3*c*). Compounds BAFGOH (Ahn *et al.*, 2015 ▸), BAFJUQ (Ahn *et al.*, 2015 ▸), VANVOX (Hilliard *et al.*, 2012 ▸) and XETSUK (Čermák *et al.*, 2001 ▸) belong to type *a* [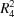
(10)]; compounds EKULUR (Chandrasekaran *et al.*, 2002 ▸), TPPOPH (Thierbach *et al.*, 1980 ▸) and UKEFEV (Sevcik *et al.*, 2003 ▸) represent type *b* [*D*_2_^2^(6)]. Finally, the title compound and DATHIQ (Ravikumar *et al.*, 2005 ▸) are of type *c* [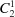
(5)]. The relative simplicity of these motifs is caused by the absence of active H atoms in coformers of the aforementioned compounds. The special case is the three-dimensional structure of tri­ethyl­enedi­amine *N*,*N*′-dioxide triperoxosolvate (FURFIH; Kay Hon & Mak, 1987 ▸).

## Synthesis and crystallization   

Needle-shaped crystals of the title compound crystallized spontaneously from a saturated solution of di­benzyl­amine in 50% hydrogen peroxide after holding for 3 d at room temperature. **Caution!** Handling procedures for concentrated hydrogen peroxide (danger of explosion) are described in detail by Wolanov *et al.* (2010 ▸).

## Refinement   

Crystal data, data collection and structure refinement details are summarized in Table 2 ▸. Peroxide H atoms were found from a difference electron-density map and refined with individual isotropic displacement parameters and restrained O—H distances. All other H atoms were placed in calculated positions, with C—H = 0.95 (aromatic) or 0.99 Å (methyl­ene), and treated as riding atoms, with relative isotropic displacement parameters *U*_iso_(H) = 1.2*U*_eq_(C).

## Supplementary Material

Crystal structure: contains datablock(s) I, global. DOI: 10.1107/S2056989017014499/zs2391sup1.cif

Structure factors: contains datablock(s) I. DOI: 10.1107/S2056989017014499/zs2391Isup2.hkl

Supporting information file. DOI: 10.1107/S2056989017014499/zs2391Isup3.cml

CCDC reference: 1578615

Additional supporting information:  crystallographic information; 3D view; checkCIF report

## Figures and Tables

**Table 1:** Hydrogen-bond geometry (Å, °)

*D*—H⋯*A*	*D*—H	H⋯*A*	*D*⋯*A*	*D*—H⋯*A*
O1—H1⋯O3	1.05 (5)	1.66 (5)	2.707 (5)	174 (4)
O2—H2⋯O3^i^	1.06 (5)	1.64 (5)	2.681 (5)	166 (4)
C21—H21⋯O1^ii^	0.95	2.46	3.304 (6)	148
C27—H27⋯O3	0.95	2.29	2.902 (6)	121
C11—H111⋯O1^ii^	0.99	2.44	3.364 (7)	155
C11—H111⋯O2^ii^	0.99	2.47	3.394 (7)	155
C11—H112⋯O2	0.99	2.52	3.407 (7)	149

Symmetry codes: (i) 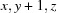; (ii) 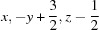.

**Table 2:** Experimental details

Crystal data	
Chemical formula	C_14_H_13_NO·H_2_O_2_
*M*_r_	245.27
Crystal system, space group	Monoclinic, *P*2_1_/*c*
Temperature (K)	150
*a*, *b*, *c* (Å)	21.802 (15), 4.597 (3), 12.742 (9)
β (°)	97.598 (11)
*V* (Å^3^)	1265.8 (16)
*Z*	4
Radiation type	Mo *K*α
μ (mm^−1^)	0.09
Crystal size (mm)	0.40 × 0.04 × 0.04

Data collection	
Diffractometer	Bruker *SMART* APEXII area-detector
Absorption correction	Multi-scan (*SADABS*; Bruker, 2008 ▸)
*T*_min_, *T*_max_	0.965, 0.996
No. of measured, independent and observed [*I* > 2σ(*I*)] reflections	7458, 2227, 1113
*R*_int_	0.108
(sin θ/λ)_max_ (Å^−1^)	0.596

Refinement	
*R*[*F* ^2^ > 2σ(*F* ^2^)], *wR*(*F* ^2^), *S*	0.077, 0.218, 1.05
No. of reflections	2227
No. of parameters	172
No. of restraints	1
H-atom treatment	H atoms treated by a mixture of independent and constrained refinement
Δρ_max_, Δρ_min_ (e Å^−3^)	0.27, −0.26

Computer programs: *APEX2* (Bruker, 2008 ▸), *SAINT* (Bruker, 2008 ▸), *SHELXS97* (Sheldrick, 2008 ▸), *SHELXL97* (Sheldrick, 2008 ▸), *SHELXTL* (Sheldrick, 2008 ▸).

## supplementary crystallographic information

### Crystal data

**Table:**

C_14_H_13_NO·H_2_O_2_	*F*(000) = 520
*M**_r_* = 245.27	*D*_x_ = 1.287 Mg m^−^^3^
Monoclinic, *P*2_1_/*c*	Mo *K*α radiation, λ = 0.71073 Å
Hall symbol: -P 2ybc	Cell parameters from 737 reflections
*a* = 21.802 (15) Å	θ = 3.2–21.9°
*b* = 4.597 (3) Å	µ = 0.09 mm^−^^1^
*c* = 12.742 (9) Å	*T* = 150 K
β = 97.598 (11)°	Needle, colourless
*V* = 1265.8 (16) Å^3^	0.40 × 0.04 × 0.04 mm
*Z* = 4	

### Data collection

**Table:**

Bruker SMART APEXII area-detector diffractometer	2227 independent reflections
Radiation source: fine-focus sealed tube	1113 reflections with *I* > 2σ(*I*)
Graphite monochromator	*R*_int_ = 0.108
ω scans	θ_max_ = 25.1°, θ_min_ = 1.9°
Absorption correction: multi-scan (SADABS; Bruker, 2008)	*h* = −25→25
*T*_min_ = 0.965, *T*_max_ = 0.996	*k* = −5→5
7458 measured reflections	*l* = −15→14

### Refinement

**Table:**

Refinement on *F*^2^	Secondary atom site location: difference Fourier map
Least-squares matrix: full	Hydrogen site location: mixed
*R*[*F*^2^ > 2σ(*F*^2^)] = 0.077	H atoms treated by a mixture of independent and constrained refinement
*wR*(*F*^2^) = 0.218	*w* = 1/[σ^2^(*F*_o_^2^) + (0.1*P*)^2^] where *P* = (*F*_o_^2^ + 2*F*_c_^2^)/3
*S* = 1.05	(Δ/σ)_max_ < 0.001
2227 reflections	Δρ_max_ = 0.27 e Å^−^^3^
172 parameters	Δρ_min_ = −0.26 e Å^−^^3^
1 restraint	Extinction correction: SHELXL97 (Sheldrick, 2008), Fc^*^=kFc[1+0.001xFc^2^λ^3^/sin(2θ)]^-1/4^
Primary atom site location: structure-invariant direct methods	Extinction coefficient: 0.034 (6)

### Special details

**Table:**

Geometry. All esds (except the esd in the dihedral angle between two l.s. planes) are estimated using the full covariance matrix. The cell esds are taken into account individually in the estimation of esds in distances, angles and torsion angles; correlations between esds in cell parameters are only used when they are defined by crystal symmetry. An approximate (isotropic) treatment of cell esds is used for estimating esds involving l.s. planes.
Refinement. Refinement of F^2^ against ALL reflections. The weighted R-factor wR and goodness of fit S are based on F^2^, conventional R-factors R are based on F, with F set to zero for negative F^2^. The threshold expression of F^2^ > 2sigma(F^2^) is used only for calculating R-factors(gt) etc. and is not relevant to the choice of reflections for refinement. R-factors based on F^2^ are statistically about twice as large as those based on F, and R- factors based on ALL data will be even larger.

### Fractional atomic coordinates and isotropic or equivalent isotropic displacement parameters (Å^2^)

**Table:**

	*x*	*y*	*z*	*U*_iso_*/*U*_eq_	
O1	0.23321 (15)	0.7522 (7)	1.0077 (3)	0.0427 (9)	
O2	0.28942 (16)	0.9116 (7)	0.9911 (3)	0.0442 (10)	
O3	0.22608 (15)	0.3093 (6)	0.8666 (2)	0.0377 (9)	
N1	0.23366 (18)	0.3713 (7)	0.7676 (3)	0.0319 (10)	
C11	0.2883 (2)	0.5529 (9)	0.7569 (4)	0.0342 (12)	
H112	0.2894	0.7210	0.8057	0.041*	
H111	0.2855	0.6284	0.6836	0.041*	
C12	0.3463 (2)	0.3775 (9)	0.7821 (4)	0.0344 (12)	
C13	0.3802 (2)	0.3866 (10)	0.8826 (4)	0.0438 (13)	
H13	0.3672	0.5083	0.9356	0.053*	
C14	0.4332 (2)	0.2183 (12)	0.9054 (5)	0.0573 (16)	
H14	0.4563	0.2229	0.9741	0.069*	
C15	0.4521 (3)	0.0431 (12)	0.8270 (5)	0.0592 (17)	
H15	0.4888	−0.0694	0.8422	0.071*	
C16	0.4190 (2)	0.0295 (12)	0.7281 (5)	0.0521 (15)	
H16	0.4317	−0.0952	0.6756	0.063*	
C17	0.3671 (2)	0.1997 (10)	0.7062 (4)	0.0423 (13)	
H17	0.3447	0.1954	0.6370	0.051*	
C21	0.1988 (2)	0.2713 (9)	0.6851 (4)	0.0334 (11)	
H21	0.2083	0.3366	0.6184	0.040*	
C22	0.1470 (2)	0.0714 (9)	0.6825 (4)	0.0330 (12)	
C23	0.1155 (2)	0.0141 (11)	0.5825 (4)	0.0432 (13)	
H23	0.1280	0.1064	0.5221	0.052*	
C24	0.0658 (2)	−0.1774 (11)	0.5704 (4)	0.0480 (14)	
H24	0.0438	−0.2129	0.5022	0.058*	
C25	0.0486 (2)	−0.3157 (11)	0.6579 (4)	0.0479 (14)	
H25	0.0149	−0.4481	0.6499	0.057*	
C26	0.0800 (2)	−0.2629 (11)	0.7565 (4)	0.0443 (14)	
H26	0.0681	−0.3606	0.8164	0.053*	
C27	0.1284 (2)	−0.0708 (9)	0.7696 (4)	0.0356 (12)	
H27	0.1495	−0.0341	0.8384	0.043*	
H1	0.232 (2)	0.588 (9)	0.950 (4)	0.060 (15)*	
H2	0.268 (2)	1.058 (10)	0.933 (4)	0.057 (15)*	

### Atomic displacement parameters (Å^2^)

**Table:**

	*U*^11^	*U*^22^	*U*^33^	*U*^12^	*U*^13^	*U*^23^
O1	0.054 (2)	0.0379 (19)	0.038 (2)	−0.0100 (17)	0.0135 (17)	−0.0039 (15)
O2	0.050 (2)	0.0394 (19)	0.044 (2)	−0.0075 (17)	0.0069 (18)	−0.0001 (16)
O3	0.052 (2)	0.0361 (17)	0.026 (2)	−0.0051 (15)	0.0106 (16)	0.0026 (14)
N1	0.041 (2)	0.027 (2)	0.029 (2)	0.0001 (18)	0.012 (2)	−0.0011 (17)
C11	0.041 (3)	0.033 (2)	0.029 (3)	−0.005 (2)	0.008 (2)	−0.001 (2)
C12	0.038 (3)	0.030 (2)	0.036 (3)	−0.007 (2)	0.006 (2)	0.004 (2)
C13	0.043 (3)	0.045 (3)	0.044 (4)	0.000 (3)	0.005 (3)	0.005 (2)
C14	0.047 (3)	0.064 (4)	0.058 (4)	0.003 (3)	−0.002 (3)	0.019 (3)
C15	0.052 (4)	0.048 (3)	0.080 (5)	0.004 (3)	0.020 (4)	0.016 (3)
C16	0.044 (3)	0.054 (3)	0.060 (4)	0.004 (3)	0.014 (3)	0.006 (3)
C17	0.042 (3)	0.043 (3)	0.043 (3)	−0.002 (3)	0.012 (3)	0.004 (2)
C21	0.038 (3)	0.031 (2)	0.031 (3)	0.004 (2)	0.001 (2)	0.001 (2)
C22	0.035 (3)	0.034 (2)	0.030 (3)	0.004 (2)	0.007 (2)	0.000 (2)
C23	0.046 (3)	0.047 (3)	0.035 (3)	0.000 (3)	0.004 (3)	−0.003 (2)
C24	0.039 (3)	0.056 (3)	0.046 (4)	−0.005 (3)	−0.005 (3)	−0.007 (3)
C25	0.042 (3)	0.051 (3)	0.053 (4)	−0.008 (3)	0.016 (3)	−0.013 (3)
C26	0.051 (3)	0.042 (3)	0.043 (3)	−0.002 (3)	0.017 (3)	−0.005 (2)
C27	0.033 (3)	0.038 (3)	0.037 (3)	−0.001 (2)	0.009 (2)	−0.004 (2)

### Geometric parameters (Å, º)

**Table:**

O1—O2	1.467 (4)	C16—C17	1.375 (7)
O1—H1	1.05 (4)	C16—H16	0.9500
O2—H2	1.06 (4)	C17—H17	0.9500
O3—N1	1.325 (4)	C21—C22	1.454 (6)
N1—C21	1.297 (6)	C21—H21	0.9500
N1—C11	1.475 (6)	C22—C23	1.390 (6)
C11—C12	1.498 (6)	C22—C27	1.393 (6)
C11—H112	0.9900	C23—C24	1.388 (7)
C11—H111	0.9900	C23—H23	0.9500
C12—C17	1.388 (6)	C24—C25	1.378 (7)
C12—C13	1.392 (7)	C24—H24	0.9500
C13—C14	1.388 (7)	C25—C26	1.371 (7)
C13—H13	0.9500	C25—H25	0.9500
C14—C15	1.389 (8)	C26—C27	1.368 (7)
C14—H14	0.9500	C26—H26	0.9500
C15—C16	1.368 (8)	C27—H27	0.9500
C15—H15	0.9500		
			
O2—O1—H1	102 (3)	C17—C16—H16	120.6
O1—O2—H2	97 (3)	C16—C17—C12	122.0 (5)
C21—N1—O3	124.2 (4)	C16—C17—H17	119.0
C21—N1—C11	121.3 (4)	C12—C17—H17	119.0
O3—N1—C11	114.4 (4)	N1—C21—C22	127.8 (4)
N1—C11—C12	110.1 (3)	N1—C21—H21	116.1
N1—C11—H112	109.6	C22—C21—H21	116.1
C12—C11—H112	109.6	C23—C22—C27	118.8 (4)
N1—C11—H111	109.6	C23—C22—C21	115.4 (4)
C12—C11—H111	109.6	C27—C22—C21	125.8 (5)
H112—C11—H111	108.1	C24—C23—C22	120.3 (5)
C17—C12—C13	118.4 (5)	C24—C23—H23	119.8
C17—C12—C11	120.8 (5)	C22—C23—H23	119.8
C13—C12—C11	120.9 (4)	C25—C24—C23	119.6 (5)
C14—C13—C12	120.2 (5)	C25—C24—H24	120.2
C14—C13—H13	119.9	C23—C24—H24	120.2
C12—C13—H13	119.9	C26—C25—C24	120.2 (5)
C13—C14—C15	119.3 (6)	C26—C25—H25	119.9
C13—C14—H14	120.3	C24—C25—H25	119.9
C15—C14—H14	120.3	C27—C26—C25	120.6 (5)
C16—C15—C14	121.3 (6)	C27—C26—H26	119.7
C16—C15—H15	119.4	C25—C26—H26	119.7
C14—C15—H15	119.4	C26—C27—C22	120.4 (5)
C15—C16—C17	118.8 (5)	C26—C27—H27	119.8
C15—C16—H16	120.6	C22—C27—H27	119.8
			
C21—N1—C11—C12	−103.9 (5)	C11—N1—C21—C22	174.4 (4)
O3—N1—C11—C12	72.7 (4)	N1—C21—C22—C23	176.4 (4)
N1—C11—C12—C17	82.7 (5)	N1—C21—C22—C27	−6.0 (7)
N1—C11—C12—C13	−96.6 (5)	C27—C22—C23—C24	1.2 (7)
C17—C12—C13—C14	−0.7 (7)	C21—C22—C23—C24	179.0 (4)
C11—C12—C13—C14	178.7 (4)	C22—C23—C24—C25	−1.4 (7)
C12—C13—C14—C15	0.5 (7)	C23—C24—C25—C26	0.5 (7)
C13—C14—C15—C16	−1.1 (8)	C24—C25—C26—C27	0.6 (7)
C14—C15—C16—C17	1.8 (8)	C25—C26—C27—C22	−0.8 (7)
C15—C16—C17—C12	−2.0 (7)	C23—C22—C27—C26	−0.1 (7)
C13—C12—C17—C16	1.4 (7)	C21—C22—C27—C26	−177.6 (4)
C11—C12—C17—C16	−178.0 (4)	H1—O1—O2—H2	88 (4)
O3—N1—C21—C22	−1.8 (7)		

### Hydrogen-bond geometry (Å, º)

**Table:**

*D*—H···*A*	*D*—H	H···*A*	*D*···*A*	*D*—H···*A*
O1—H1···O3	1.05 (5)	1.66 (5)	2.707 (5)	174 (4)
O2—H2···O3^i^	1.06 (5)	1.64 (5)	2.681 (5)	166 (4)
C21—H21···O1^ii^	0.95	2.46	3.304 (6)	148
C27—H27···O3	0.95	2.29	2.902 (6)	121
C11—H111···O1^ii^	0.99	2.44	3.364 (7)	155
C11—H111···O2^ii^	0.99	2.47	3.394 (7)	155
C11—H112···O2	0.99	2.52	3.407 (7)	149

Symmetry codes: (i) *x*, *y*+1, *z*; (ii) *x*, −*y*+3/2, *z*−1/2.
